# A phase 1b study of Selumetinib in combination with Cisplatin and Gemcitabine in advanced or metastatic biliary tract cancer: the ABC-04 study

**DOI:** 10.1186/s12885-016-2174-8

**Published:** 2016-02-24

**Authors:** John Bridgewater, Andre Lopes, Sandra Beare, Marian Duggan, Dymphna Lee, Maravic Ricamara, Delyth McEntee, Ajithkumar Sukumaran, Harpreet Wasan, Juan W. Valle

**Affiliations:** UCL Cancer Institute, 72 Huntley St, London, WC1E 6DD UK; Cancer Research UK and UCL Clinical Trials Centre, London, UK; The Christie NHS Foundation Trust, Manchester, UK; Imperial College Healthcare, London, UK

**Keywords:** Biliary tract cancer, Selumetinib, Cisplatin, Gemcitabine, Phase 1b

## Abstract

**Background:**

Combined treatment with cisplatin and gemcitabine (CisGem) is the standard of care for patients with advanced biliary tract cancer (ABC). Selumetinib (AZD6244, ARRY-142886) potently and selectively inhibits MEK1/2, an intracellular kinase and has shown activity in ABC. The objective of the ABC-04 trial was to establish the recommended dose of selumetinib in combination with CisGem in patients with ABC.

**Methods:**

Eligible patients were ≥ 18 years, had histologically or cytologically-confirmed unresectable recurrent or metastatic biliary tract, gallbladder or ampullary carcinoma, WHO performance status 0–2, and adequate major organ function. Patients may have had prior surgery, radiotherapy or adjuvant chemotherapy, but no prior CisGem and no prior chemotherapy for locally advanced or metastatic disease. Patients received cisplatin 25 mg/m^2^ plus gemcitabine 1000 mg/m^2^ intravenously on days 1 and 8 of a 21-day cycle. Selumetinib capsules were taken daily. Patients received up to 8 cycles of CisGem and could receive selumetinib until disease progression. A dose de-escalation scheme was used to determine the recommended dose of selumetinib. The first dose level was 75 mg bd. Patients were recruited in cohorts of 3 and assessed for dose limiting toxicity (DLT) during the first cycle of treatment.

**Results:**

Thirteen patients were recruited, of whom 12 were evaluable for DLT (1 did not start treatment). All evaluable patients received the starting dose of selumetinib 75 mg bd and one patient experienced a DLT (cardiac chest pain). The median number of days selumetinib was taken (adjusted for the number of days of dose interruptions) was 171.5 (IQR: 75.5 to 344). Two patients remained on treatment at 14 and 19 months post registration. There were 3 temporary and 1 permanent interruptions of selumetinib in cycle 1. Eight patients were evaluable for objective response (RECIST v1.1): 3 had a partial response and 5 stable disease. The median PFS was 6.4 months (IQR 5.2 to 13.7). Toxicities related to selumetinib were mostly related to oedema and rash, grade 1–2 and manageable. Pharmacokinetic analysis showed that the AUC(0-t), AUC(0-∞) and Cmax of selumetinib increased by 12, 11 and 30 % respectively when it was administered with CisGem, while Cmax for the N-desmethyl metabolite of selumetinib decreased by 40 %. There was no evidence that the time of Cmax for selumetinib or N-desmethyl metabolite of selumetinib were different when selumetinib was administered alone or with CisGem.

**Conclusion:**

The recommended dose of selumetinib when combined with CisGem was 75 mg bd. Translational studies are underway to identify biomarkers that may predict outcome (ClinicalTrials.gov identifier: NCT01242605 July 6^th^ 2010).

**Electronic supplementary material:**

The online version of this article (doi:10.1186/s12885-016-2174-8) contains supplementary material, which is available to authorized users.

## Background

Biliary tract cancer is an uncommon cancer in developed countries with approximately 1200 new cases in the UK and 9000 new cases in the United States per year. The incidence is increasing, perhaps related to gall stone disease. Most patients have advanced disease at presentation, or relapse after surgery for localised disease. The National Cancer Research Institute (UK) ABC-02 study demonstrated the combination of cisplatin and gemcitabine to be an effective first line treatment and this combination has become the international standard of care [1].

Selumetinib (AZD6244, ARRY-142886) is a potent and selective inhibitor of MEK [MAPK (mitogen-activated protein kinase)-ERK (extracellular-signal-regulated kinase) kinase]. Selumetinib inhibits phosphorylation of ERK2 by MEK and decreased levels of phosporylated ERK (pERK) post treatment have been shown to correlate with efficacy [2, 3]. Mutations in the genes of downstream signaling molecules such as KRAS and BRAF have been found to be associated with resistance to cetuximab but these effects are, to a certain extent, tissue and context dependent [4, 5].

In the clinic, selumetinib 75 mg twice daily (bd) has been tolerated in combination with docetaxel or dacarbazine in phase II studies [6, 7]. Bekaii-Saab and colleagues [8] conducted a phase II trial of selumetinib monotherapy at 100 mg bd in 29 patients with ABC, 39 % of whom had received prior chemotherapy. An older formulation of selumetinib was used in Bekaii-Saab’s trial and the exposure from the 100 mg dose would have been similar to the 75 mg dose of the new formulation used in ABC-04. The response rate was 12 % which is comparable with single agent gemcitabine in this setting. Decreased levels of pERK were associated with selumetenib as well as improved progression free survival and putative effects on muscle bulk were also observed [9].

In summary, selumetinib has shown promise in ABC as a single agent and has been safely combined with chemotherapy. These data suggested that selumetinib may be effective in combination with CisGem for patients with ABC. We performed a phase Ib study of the CisGem + selumetinib combination to determine the recommended dose of selumetinib for subsequent efficacy-based studies. Secondary objectives were to investigate pharmacokinetic (PK) interactions between selumetinib and gemcitabine, to determine the objective tumour response rate (ORR) by CT or MRI, and to determine time to progression or death from any cause (PFS and OS).

## Methods

### Trial design and treatment

ABC-04 was a phase Ib, multicentre, open-label trial which used a dose de-escalation strategy to determine the recommended dose of selumetinib in combination with CisGem (ClinicalTrials.gov identifier: NCT01242605). A dose de-escalation strategy was chosen since previous trials had shown that 75 mg bd selumetinib could be given safely with chemotherapy; namely, docetaxel [6] and dacarbazine [7]. Four dose levels were included in the protocol for potential evaluation; 75 mg bd, 50 mg bd, 75 mg once daily (od) and 50 mg od. The first dose level evaluated was 75 mg bd. Patients were recruited in cohorts of three. The number of patients experiencing a DLT during the first cycle of treatment in each cohort was used to decide the dose level at which to recruit the next cohort. If no patients experienced a DLT, then that dose level was the recommended dose. If one patient experienced a DLT, the cohort was to be expanded to six patients. If > 2 patients experienced DLT, then subsequent patients were to be entered into the next lower dose level. A minimum of 12 patients were to be treated at the recommended dose to further establish safety.

Chemotherapy consisted of cisplatin 25 mg/m^2^ plus gemcitabine 1000 mg/m^2^ administered intravenously (IV) on days 1 and 8 of a 21-day cycle for up to 8 cycles, as previously described [1]. Pre-chemotherapy hydration and anti-emetic regimens were as per each site’s policy. Selumetinib was taken orally each day throughout chemotherapy. Three consecutive days of selumetinib were administered prior to the first cycle of chemotherapy in order to obtain PK data for selumetinib alone. Patients could continue with selumetinib after completion of chemotherapy until disease progression if it was deemed to be in the best interests of the patient.

The trial was reviewed and approved by the South Central-Berkshire Research Ethics Committee and the Medicines and Healthcare Products Regulatory Authority. Global National Health Service (NHS) permission was granted by the Central and East London Comprehensive Local Research Network (CLRN), and the trial was approved by the Research and Development department of each participating NHS Trust. The trial was performed in accordance with the principles of International Conference on Harmonisation Good Clinical Practice Guidelines. It was coordinated by the Cancer Research UK and University College London Cancer Trials Centre and sponsored by University College London. The views expressed are those of the authors and not necessarily those of the NHS, the NIHR or the Department of Health.

### Patients

Patients were eligible if they had a histopathological or cytological diagnosis of non-resectable, recurrent or metastatic biliary tract (intra- or extra-hepatic), gallbladder or ampullary carcinoma for which no prior systemic chemotherapy had been given. Patients were required to have World Health Organisation (WHO) performance status ≤ 2, be aged ≥ 18, have an estimated life expectancy > 3 months, and have adequate biliary drainage and major organ function. Patients had to be able to swallow selumetinib capsules and be capable of giving written informed consent. Patients were not eligible if they had had any prior exposure to MEK, RAS, or RAF inhibitors, had not fully recovered from previous surgery, had had a prior malignancy (except curatively treated in-situ carcinoma of the cervix, non-metastatic basal and/or squamous cell carcinomas of the skin, or any early stage (stage I) malignancy adequately resected for cure greater than 5 years previously), had evidence of a severe or uncontrolled systemic disease, if the patient was pregnant or breast-feeding, or had any of the following cardiac conditions: uncontrolled hypertension (blood pressure ≥150/95 despite optimal therapy), heart failure (New York Heart Association Class II or above), prior or current cardiomyopathy, baseline left ventricular ejection fraction ≤ 50 %, atrial fibrillation with heart rate >100 beats per minute, unstable ischaemic heart disease (myocardial infarction within 6 months prior to starting treatment, or angina requiring use of nitrates more than once weekly).

All patients gave written informed consent prior to any trial-specific interventions.

### Assessments

Patients were assessed prior to starting treatment and at regular intervals throughout treatment. Physical examinations, including vital signs and assessment of adverse events (AEs) were carried out prior to registration, on days 1 and 8 of each cycle of chemotherapy and monthly for patients who continued on selumetinib alone. All AEs occurring between the date of signing consent and 30 days after the last dose of any trial treatment were recorded. AEs were graded according to CTCAE v 4.03. Blood biochemistry and a full blood count with differential were carried out at the same time points as the physical examinations. Patients who had a calculated creatinine clearance of < 45 mL/min prior to treatment were required to have an isotopic glomerular filtration rate (GFR) test to confirm GFR > 45 mL/min. In addition, prior to registration, an electrocardiogram and echocardiogram, and an ophthalmological examination were required. Disease status was assessed by computed tomography (CT) scan, with or without a liver magnetic resonance (MR) scan, prior to registration, after cycles 4 and 8 and then subsequently every 12 weeks until disease progression. Patients with measurable disease at baseline were assessed for objective response according to RECIST v1.1 [10]. For patients with non-measurable disease, objective response was not assessed and progressive disease was documented by the appearance of new lesions or by symptomatic deterioration.

### Dose limiting toxicity

Patients were assessed for DLT during the first cycle of treatment. Patients experiencing any of the following drug-related adverse events were considered to have experienced a DLT: Any grade 3 or 4 AE or laboratory abnormality that was deemed clinically significant by the investigator; infusion-related reactions were considered a DLT based on the study team’s medical review of the event; grade 3 fatigue that persisted for > 7 days, or grade 4 fatigue; grade 3 or 4 nausea, diarrhoea, or vomiting despite maximum supportive care; magnesium <0.4 mmol/L; grade 3 rash/desquamation for > 7 days; grade 3 or 4 neutropenia with fever ≥ 38.5 °C lasting 3 days or more; grade 4 neutropenia or thrombocytopenia > 7 days; ALT or AST > 10 x ULN; patient was unable to tolerate a total of at least one three week dosing course due to adverse event(s); any AE resulting in a more than 14 day treatment delay for any agent. To be evaluable for evaluation of the recommended dose, the patient must have completed 21 days (3 weeks or 1 cycle) of therapy must and received 80 % of expected dose of all drugs, or the patient must have experienced a DLT during cycle 1. Patients who did not meet these criteria were replaced.

### Pharmacokinetics

Blood samples were collected from all patients entering the study for the determination of plasma drug and metabolite concentrations for selumetinib. Cisplatin pharmacokinetics were not analysed as an interaction with selumetinib was unlikely since the metabolic pathways are different. Samples were taken for analysis of the effect of selumetinib on gemcitabine PK. However, the gemcitabine sample collection was incomplete therefore only selumetinib PK are presented.

Blood samples for selumetinib PK analysis were collected at the following time points: before the start of trial treatment; on the third day of selumetinib dosing (selumetinib alone) just before the morning dose and after the morning dose at 15 min, 30 min, 1 h, 1.5 h, 2 h, 4 h, 8 h, 10 h (before the evening dose of selumetinib for bd dosing), and 24 h (before the morning dose of selumetinib and start of chemotherapy); On cycle 1 day 8 (selumetinib + CisGem) before the morning dose of selumetinib and before chemotherapy and after the morning dose of selumetinib at 15 min, 30 min, 1 h (at end of cisplatin infusion), 1.5 h, 2 h (at end of gemcitabine infusion), 4 h, 8 h, 10 h (before the evening dose of selumetinib for bd dosing), and 24 h (before the morning dose of selumetinib).

Where data allowed, the following PK parameters were determined following administration selumetinib alone and following administration of selumetinib and CisGem together. For selumetinib alone, maximum observed concentration (C_max_), time to C_max_ (t_max_), area under the curve (AUC)_(0-12)_ and apparent oral clearance (CL/F, Apparent oral clearance = (dose*F)/AUC where F = absolute bioavailability of the drug), and when given in combination, C_max_ ratio, AUC_(0-12)_ ratio. For N-desmethyl selumetinib when selumetinib was given alone, C_max_, t_max_, AUC_(0-12),_ AUC_(0-t),_ CL/F, metabolite:parent ratio for C_max_ and AUC_(0-12),_ and given in combination with CisGem: C_max_ ratio, AUC ratio.

### Other statistical analyses

Tumour responses were assessed according to RECIST version 1.1. The best response across all time points has been reported. For PFS and OS, the time to progression or death was measured from the date of registration. PFS and OS were estimated for the population by the Kaplan-Meier method.

All safety data was summarized by frequency and percentage and presented for each dose as appropriate. Worst grade per patient has been reported.

## Results

### Recruitment information

**Fig. 1:**
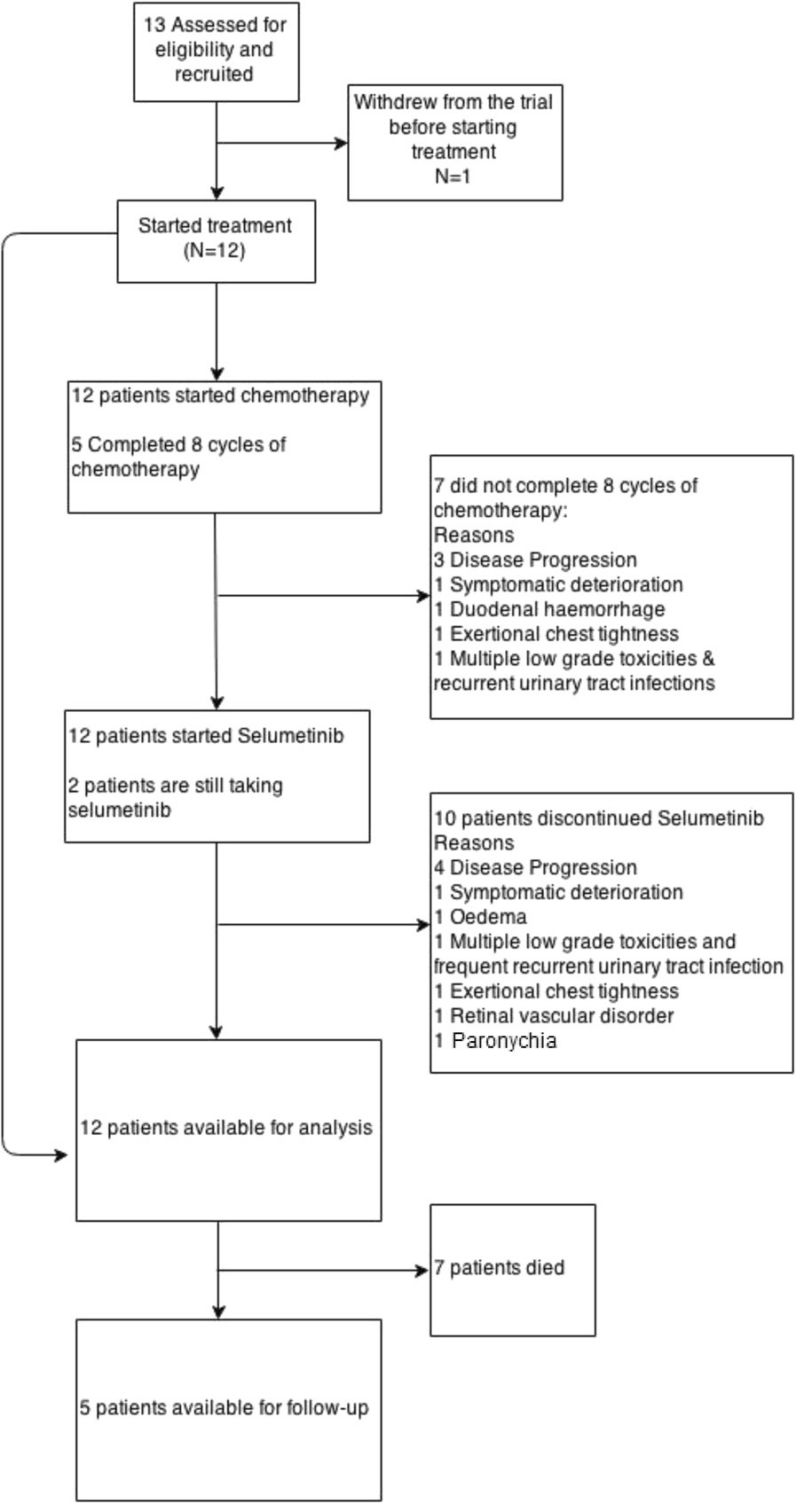
Consort diagram for the ABC-04 study

**Fig. 2:**
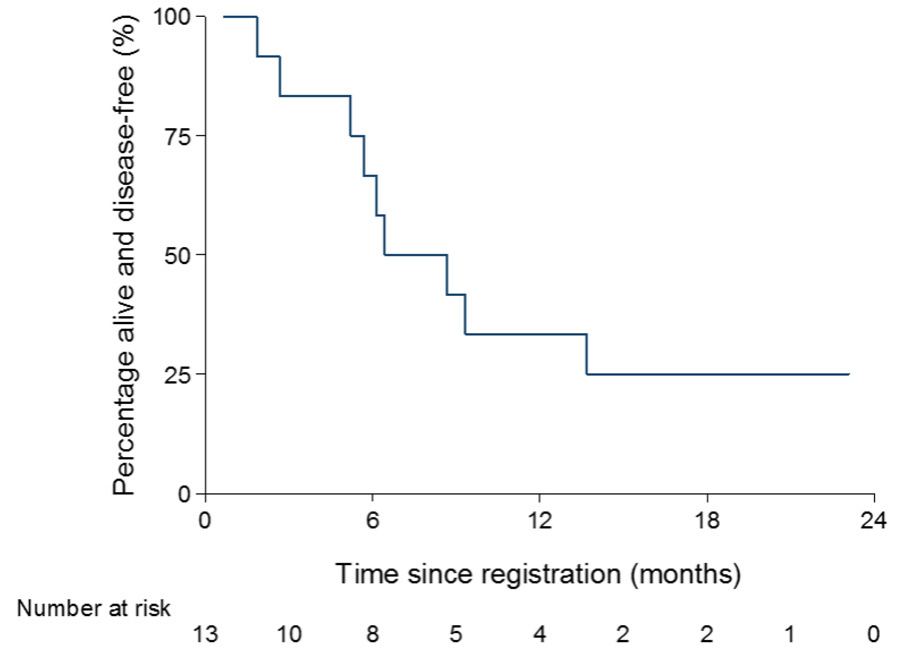
Kaplan Meier curve of progression free survival

Thirteen patients were recruited from 3 centres in the United Kingdom between February 2012 and January 2013 of whom 12 were evaluable for DLT (Fig. 1). The median follow-up time was 20.4 months using censored deaths method. The data cut off was 28^th^ April 2014 (Fig. 2).

### Baseline characteristics

Patient demographics are given in Table 1. The median age was 65 years (range: 45–81 years); 9 patients (69 %) had bile duct as the primary site of disease, 3 (23 %) had gall bladder cancer and 1 (8 %) had ampullary cancer. The advanced stage of patients’ disease was evident by high T stage (T3-4) in 70 %, LN positive disease in 46 % and the presence of metastases in 77 % of patients. A total of 8 patients (62 %) had previous surgery and 7 (54 %) had biliary stent insertions.

**Table 1:** Baseline characteristics

Variables	Number of patients (%)
Age (years)	
Median (IQR)	65 (61 to 68)
Sex	
Female	9 (69 %)
Male	4 (31 %)
Primary site	
Gall bladder	3 (23 %)
Bile duct	9 (69 %)
Ampulla	1 (8 %)
ECOG PS	
0	9 (69 %)
1	4 (31 %)
T-stage	
T1	1 (8 %)
T2	0 (0 %)
T3	8 (62 %)
T4	1 (8 %)
TX	3 (23 %)
N-stage	
N0	2 (15 %)
N1	5 (38 %)
N2	1 (8 %)
NX	5 (38 %)
M-stage	
M0	1 (8 %)
M1^a^	10 (77 %)
MX	2 (15 %)
Tumour differentiation	
Well/Moderately differentiated	6 (46 %)
Poorly differentiated	6 (46 %)
Not specified	1 (8 %)
Previous treatment	
None	5 (38 %)
Chemotherapy	0 (0 %)
Surgery	8 (62 %)
Radiotherapy	0 (0 %)
Other	0 (0 %)
2+ types	0 (0 %)
Biliary Stent Insertions	7 (54 %)

^a^Sites of metastasis: 8 patients had liver metastases, 3 had peritoneum, 2 had lung. Two patients had other metastasis: one had small bowel and the other had coeliac lymph node positive gastric lymph node

### Compliance with CisGem chemotherapy

One patient withdrew from the trial before starting treatment due to ill health (gallbladder perforated into the liver causing a collection requiring drainage via a cholecystostomy). Five patients (42 %) completed 8 cycles of CisGem. Of the 7 who did not complete 8 cycles of CisGem, 3 stopped early due to disease progression, 1 patient due do symptomatic deterioration and 3 patients due to unacceptable adverse events (1 had grade 3 angina/chest pain cardiac (1 cycle of chemotherapy and selumetinib), 1 had grade 3 duodenal haemorrhage (7 cycles of chemotherapy and selumetinib), and 1 had multiple low grade toxicities & recurrent urinary tract infections (4 cycles of chemotherapy and selumetinib)).

### Compliance with selumetinib

The median length of time selumetinib was taken was 171.5 days (IQR: 75.5 to 344). Four selumetinib dosing suspensions occurred during cycle 1 of chemotherapy. One patient stopped selumetinib permanently during cycle 1 due to grade 3 cardiac chest pain, and this was considered to be a DLT. Three patients each had one temporary suspension of selumetinib during cycle 1 all of which were less than 14 days (blurred vision, dyspnoea and chest pain, and vomiting and depressive symptoms). At the time of the final analysis, ten patients had stopped selumetinib permanently: 4 (40 %) due to adverse events (see Additional file 1: Table S2) and 5 (50 %) due to disease progression or symptomatic deterioration. All 12 patients had at least one temporary suspension of selumetinib at some point during their time on study treatment.

### Dose limiting toxicities

Only one patient experienced a DLT which was grade 3 cardiac chest pain requiring permanent discontinuation of protocol treatment during cycle 1. No dose de-escalation step for selumetinib was required. Toxicity associated with the selumetinib dose of 75 mg bd was manageable.

### Toxicity

General infection, raised gamma-glutamyl transpeptidase (GGT) and low neutrophil count were the most common grade 3 and 4 adverse events. Grade 3 or 4 toxicities considered to be related to the administration of selumetinib occurred in 25 % of patients (Table 2). Three patients (25 %) developed grade 3 or 4 infections considered to be related to selumetinib: 2 patients had a skin infection and 1 patient had a bronchial infection. Relatedness to selumetinib is given in Additional file 2: Table S1.

**Table 2:** Adverse events (worst grade during the trial)

Adverse events (CTCAE v4.03) ^a,b^	Worst grade		
	Grades 1 & 2	Grades 3 & 4	Any Grade
	N(%)	N(%)	N(%)
Haemotological			
Neutropenia	6 (50 %)	1 (8 %)	7 (58 %)
Platelets	5 (42 %)	3 (25 %)	8 (67 %)
Hb	8 (67 %)	4 (33 %)	12 (100 %)
Neutrophils	5 (42 %)	5 (42 %)	10 (83 %)
Liver function			
ALT	6 (50 %)	4 (33 %)	10 (83 %)
AST	11 (92 %)	1 (8 %)	12 (100 %)
Bilirubin	3 (25 %)	1 (8 %)	4 (33 %)
ALP	9 (75 %)	3 (25 %)	12 (100 %)
GGT	6 (50 %)	6 (50 %)	12 (100 %)
Non-haematological			
Hypertension	0 (0 %)	1 (8 %)	1 (8 %)
Lethargy	2 (17 %)	0 (0 %)	2 (17 %)
Fatigue	8 (67 %)	2 (17 %)	10 (83 %)
Rash	6 (50 %)	0 (0 %)	6 (50 %)
Anorexia	9 (75 %)	0 (0 %)	9 (75 %)
Nausea	9 (75 %)	0 (0 %)	9 (75 %)
Vomiting	9 (75 %)	0 (0 %)	9 (75 %)
Constipation	9 (75 %)	1 (8 %)	10 (83 %)
Diarrhoea	5 (42 %)	1 (8 %)	6 (50 %)
Oedema	11 (92 %)	0 (0 %)	11 (92 %)
Allergy reaction	1 (8 %)	0 (0 %)	1 (8 %)
Tinnitus	1 (8 %)	0 (0 %)	1 (8 %)
Dyspnoea	3 (25 %)	1 (8 %)	4 (33 %)
Blurred vision	4 (33 %)	0 (0 %)	4 (33 %)
Other			
Haemorrhage	2 (17 %)	1 (8 %)	3 (25 %)
Bacteraemia	1 (8 %)	0 (0 %)	1 (8 %)
Fever	7 (58 %)	0 (0 %)	7 (58 %)
Mucositis/oral thrush	8 (67 %)	0 (0 %)	8 (67 %)
Other mucositis	1 (8 %)	0 (0 %)	1 (8 %)
Paronychia	1 (8 %)	1 (8 %)	2 (17 %)
General infection	1 (8 %)	7 (58 %)	8 (67 %)
Biliary sepsis	1 (8 %)	2 (17 %)	3 (25 %)
Sensory neuropathy	5 (42 %)	0 (0 %)	5 (42 %)
Thromboembolic event	0 (0 %)	2 (17 %)	2 (17 %)
Chest pain—cardiac	1 (8 %)	1 (8 %) ^c^	2 (17 %)
Non-specific pain	9 (75 %)	1 (8 %)	10 (83 %)
Pancreatitis	1 (8 %)	0 (0 %)	1 (8 %)
Alopecia	3 (25 %)	0 (0 %)	3 (25 %)
Heart failure	0 (0 %)	1 (8 %)	1 (8 %)
Nasal discharge/congestion	4 (33 %)	0 (0 %)	4 (33 %)
Dyspepsia/Dysphagia	5 (42 %)	0 (0 %)	5 (42 %)
Retinal vascular disorder	1 (8 %)	0 (0 %)	1 (8 %)
Depressive symptoms	1 (8 %)	1 (8 %)	2 (17 %)
Palmar-plantar erythema	1 (8 %)	0 (0 %)	1 (8 %)
Other adverse events	11 (92 %)	0 (0 %)	11 (92 %)

^a^One patient can appear in more than one row

^b^Percentages are based on a total of 12 patients. One patient did not start treatment

^c^This adverse event was considered a DLT (Grade 3)

### Cause of death and PFS

At the time of the final analysis, 7 patients (59 %) had died and 5 patients (41 %) were alive. The 7 patients who died and 2 of the patients alive had progressed. Six patients died due to disease and 1 due to metabolic liver disease (primary cause) and pulmonary embolism (secondary cause). None of the deaths were considered to be related to protocol treatment. The PFS rate at 6 months was 67 % and at 12 months was 33 % and the median PFS was 6.4 months (IQR 5.2 to 13.7)

.

### Objective response

Eight patients were evaluable for objective tumour response using RECIST v1.1 (Additional file 3: Table S4). Median time from documented best objective response until progression or death is 5.9 months. The patient who had complete response has no documented progression or death. The patient who had partial response had documented progression at 5.9 months. A total out of 6 patients who had stable disease had a documented PFS event (4 had had a documented progression and death; 1 had documented death only). For patients who had best objective response SD, the median time from documented best objective response until progression or death is 3.1 months.

### PK analysis

Eleven patients were evaluable for the PK analysis (Table 3). Data from one patient was excluded from the analysis because of extensive missing data. Missing concentrations for selumetinib and N-desmethyl selumetinib (6 values, Additional file 4: Table S3) were imputed using predicted values from the concentration at the previous PK time point using linear regression. The plasma concentration of selumetinib (S) increased in the presence of CisGem (S + CisGem) compared to when selumetinib was given alone. In the presence of CisGem, the ratios of the geometric means of S + CisGem compared to S of AUC (0-t) and AUC (0-∞) of selumetinib increased by 12 % (ratio S + CisGem:S 1.12 (90 % CI: 0.95 to 1.30)) and 11 % (ratio S + CisGem:S 1.11 (90 % CI: 0.95 to 1.30)), respectively. In the presence of CisGem, the geometric mean of Cmax for selumetinib increased by 30 % (ratio S + CisGem:S 1.30 (90 % CI: 0.91 to 1.85)). There was no evidence that Tmax changed in the presence of CisGem because the median of the paired differences of Tmax for selumetinib plus CisGem and selumetinib alone was 0 (90 % CI: -1.09 to 0 .57). The geometric means of AUC(0-t), AUC (0-∞) and Cmax for the N-desmethyl metabolite of selumetinib all decreased by 40 % in the presence of CisGem. Tmax for N-desmethyl selumetinib did not change in the presence of CisGem (median of paired differences 0 (90 % CI: -1.13 to 0 .50)).

**Table 3:** Pharmacokinetic parameters of selumetinib, by treatment combinations (75 mg bd)

Compound	PK Parameters^a^	N^b^	Sel + CisGem	Selumetinib alone	Sel vs Sel + CisGem Effect (90 % CI)^e^
Selumetinib	AUC(0-t) (ng-hr/mL)	11	5304.37	4757.16	1.12 (0.95 to 1.30)
	AUC(0-∞) (ng-hr/mL)^c^	11	6286.25	5653.31	1.11 (0.95 to 1.30)
	Cmax (ng/mL)	11	1725.64	1331.33	1.30 (0.91 to 1.85)
	Tmax (hours)^d^	11	1.50	1.55	0 (−1.09 to 0 .57)
N-desmethyl selumetinib	AUC(0-t) (ng-hr/mL)	11	216.83	347.26	0.62 (0.43 to 0.91)
	AUC(0-∞) (ng-hr/mL)^c^	11	277.55	452.88	0.61 (0.42 to 0.89)
	Cmax (ng/mL)	11	48.41	77.16	0.63 (0.43 to 0.91)
	Tmax (hours)^d^	11	1.62	2.00	0 (−1.13 to 0 .50)

^a^Analysis used the actual time points when PK samples were collected. PK time point 0 corresponded to the time selumetinib was administered. Missing times of selumetinib dose administration were imputed from the non-missing time points. Missing concentrations for selumetinib and N-desmethyl selumetinib were estimated using linear interpolation

^b^Of 12 patients, 11 were evaluable for PK analysis. Patients evaluable for PK analysis included the patients for whom a PK parameter could be imputed

^c^Log linear models were used to extrapolate AUC (0-∞) using the last three measurements

^d^Tmax is expressed in terms of medians

^e^Ratio of geometric means between selumetinib and selumetinib + CisGem for AUC(0-t), AUC(0-∞) and Cmax. Rank-based difference in paired medians for Tmax

## Discussion

Although significant advances have been made in the management of patients with ABC, the prognosis remains poor. The molecular landscape of biliary tract cancers has been described although clear molecular targets for therapy remain elusive [11–16]. Clinical trials using anti-epidermal growth factor antibody in combination with oxaliplatin and gemcitabine in a randomised phase II study failed to demonstrate benefit for the experimental arm [17]. Another randomised phase II study of gemcitabine with or without sorafenib also failed to demonstrate benefit for the experimental arm [18]. The randomised phase II ABC-03 study which allocated patients to CisGem and placebo or CisGem plus cediranib showed no difference in PFS [19].

The role for targeted agents in biliary tract cancer is uncertain and benefit from these drugs is likely to require identification of molecular profiles in this patient group that predict outcome. Although preclinical data in breast and non-small cell lung cancers [20] suggest biomarkers predictive of response, there are significant differences between types of cancer and also heterogeneity within individual cancers themselves. Next Generation Sequencing data of biliary tract cancers [12, 21] suggest a disparate molecular genotype, with some studies showing a high frequency of BAP1, ARID1A and PBRM1 mutations and others TP53, KRAS and SMAD4. Further data suggest the single largest subgroup are those with amplified HER2 expression [16]. To this end, there is ongoing translational analysis of the ABC-04 material.

In ABC-04 one dose limiting toxicity was observed, which was cardiac chest pain in a patient not known to have coronary artery disease. There was a clear temporal relationship between selumetinib administration and chest pain and this has not been seen in patients treated with CisGem chemotherapy alone, including the ABC-02 study [1]. In general, the combination of CisGem + selumetinib was delivered with manageable toxicity at a dose of 75 mg bd. This was the recommended dose determined by similar studies of selumetinib-chemotherapy combinations [6, 7] and, as expected, no dose de-escalation was required in our study. The impact of CisGem on selumetinib and N-desmethyl selumetinib AUC is interesting in view of the increased potency of the metabolite and would suggest a considering a higher dose level to reach MTD in future studies. As such however, the dose level described here represents the current dose for use in combination with CisGem for future investigation.

A role for selumetinib has been defined in uveal melanoma [22], melanoma [7] and non-small cell lung cancer [6]. MEK inhibitors have also shown promise in pancreatic cancer. Analysis of the mechanisms of MEK resistance have demonstrated up-regulation of regulators in parallel pathways suggesting combinations of pathway inhibition may be required to optimally reduce the growth signal [23–25]. We conclude that selumetinib can be given with CisGem in advanced biliary tract cancer at the dose of 75 mg bd with manageable toxicity. Its role as an effective therapy will be determined by randomised phase II and III studies in the context of molecular profiling. We would propose further investigation of this combination.

## Conclusion

The recommended dose of selumetinib when combined with CisGem was 75 mg bd. Translational studies are underway to identify biomarkers that may predict outcome.

## Additional files

Additional file 1: Table S2. Permanent dose suspensions at any time point. (DOCX 12 kb) Additional file 2: Table S1. Adverse events—related with selumetinib*. (DOCX 17 kb) Additional file 3: Table S4. Best objective response (RECIST v1.1). (DOCX 11 kb) Additional file 4: Table S3. Missing timepoints. (DOCX 10 kb)

## Abbreviations

ABC: Advanced biliary tract cancer
ABC-04: Advanced biliary tract cancer Study 04
AE: Adverse events
ALT: Alanine transaminase
AST: Aspartate transaminase
AUC: Area under the curve
bd: Twice daily
CisGem: ABC-02 cisplatin gemcitabine regimen
CLRN: Comprehensive Local Research Network
CL/F: Apparent oral clearance
Cmax: Maximum concentraion
CT: Computer tomography
CTCAE: *Common terminology criteria for adverse events*
DLT: Dose limiting toxicity
ERK: Extracellular-signal-regulated kinase
GGT: Gamma-glutamyl transpeptidase
GFR: Glomerular filtration rate
IQR: Interquadrantic ratio
IV: Intravenously
MEK1/2: Mitogen-activated protein kinase kinase 1
mg/m^2^: Milligrams/metre squared
MRI: Magnetic resonance imaging
NHS: National Health Service
NIHR: National Institute for Health Research
od: Once daily
ORR: Objective tumour response rate
OS: Overall survival
pERK: Phosporylated ERK
PK: Pharmacokinetics
PFS: Progression free survival
RAF: RAF proto-oncogene
RAS: Rous Sarcoma virus proto-oncogene
RECIST: Response Evaluation Criteria in Solid Tumours
S: Selumetinib
S + CisGem: Selumetinib and CisGem
t_max_: Time to Cmax
ULN: Upper limit of normal
WHO: World health organisation
