# Erratum to: A phase 1b study of Selumetinib in combination with Cisplatin and Gemcitabine in advanced or metastatic biliary tract cancer: the ABC-04 study

**DOI:** 10.1186/s12885-016-2403-1

**Published:** 2016-06-21

**Authors:** John Bridgewater, Andre Lopes, Sandra Beare, Marian Duggan, Dymphna Lee, Maravic Ricamara, Delyth McEntee, Ajithkumar Sukumaran, Harpreet Wasan, Juan W. Valle

**Affiliations:** UCL Cancer Institute, 72 Huntley St, London, WC1E 6DD UK; Cancer Research UK and UCL Clinical Trials Centre, London, UK; The Christie NHS Foundation Trust, Manchester, UK; Imperial College Healthcare, London, UK

## Erratum

After publication of the original article [1], the authors noticed an error in the Acknowledgements section. The Acknowledgement contains missing information. The correct version of the Acknowledgements section is included below:
