# Supplementary material for: A phase 1b study of Selumetinib in combination with Cisplatin and Gemcitabine in advanced or metastatic biliary tract cancer: the ABC-04 study
Source: BMC Cancer. 2016 Feb 24;16:153. doi: 10.1186/s12885-016-2174-8 (PMC4766710; doi:10.1186/s12885-016-2174-8)
Supplement: Additional file 1: Table S2. — Permanent dose suspensions at any time point. (DOCX 12 kb) [file 12885_2016_2174_MOESM1_ESM.docx]

Supplementary table 2: **Permanent dose suspensions at any time point**

|  |  |
| --- | --- |
| *Reasons* |  |
| Disease Progression | 4 (33%) |
| Symptomatic deterioration | 1 (8%) |
| Oedema | 1 (8%) |
| Multiple low grade toxicities and frequent recurrent urinary tract infection | 1 (8%) |
| Exertional chest tightness | 1 (8%) |
| Retinal vascular disorder | 1 (8%) |
| Selumetinib discontinued for over 4 weeks | 1 (8%) |
