# Supplementary material for: A phase 1b study of Selumetinib in combination with Cisplatin and Gemcitabine in advanced or metastatic biliary tract cancer: the ABC-04 study
Source: BMC Cancer. 2016 Feb 24;16:153. doi: 10.1186/s12885-016-2174-8 (PMC4766710; doi:10.1186/s12885-016-2174-8)
Supplement: Additional file 2: Table S1. — Adverse events—related with selumetinib*. (DOCX 17 kb) [file 12885_2016_2174_MOESM2_ESM.docx]

| Table S1 - Adverse events - related with selumetinib* | | | | | |
| --- | --- | --- | --- | --- | --- |
| Adverse events  (CTCAE v4.03) ^1, 2^ | Worst grade | | | | |
|  | Grade 1 N(%) | Grade 2 N(%) | Grade 3 N(%) | Grade 4 N(%) | Any grade N(%) |
|  |  |  |  |  |  |
| Haemotological |  |  |  |  |  |
| WBC | 1 (8%) | 1 (8%) | 0 (0%) | 0 (0%) | 2 (17%) |
| Platelets | 1 (8%) | 1 (8%) | 0 (0%) | 0 (0%) | 2 (17%) |
| Hb | 2 (17%) | 1 (8%) | 0 (0%) | 0 (0%) | 3 (25%) |
| Neutrophils | 0 (0%) | 3 (25%) | 1 (8%) | 0 (0%) | 4 (33%) |
| Any haem AEs | 3 (25%) | 4 (33%) | 1 (8%) | 0 (0%) | 4 (33%) |
|  |  |  |  |  |  |
| Liver function |  |  |  |  |  |
| ALT | 1 (8%) | 0 (0%) | 1 (8%) | 1 (8%) | 3 (25%) |
| AST | 1 (8%) | 2 (17%) | 0 (0%) | 0 (0%) | 3 (25%) |
| Bilirubin | 0 (0%) | 0 (0%) | 0 (0%) | 0 (0%) | 0 (0%) |
| ALP | 0 (0%) | 1 (8%) | 2 (17%) | 0 (0%) | 3 (25%) |
| GGT | 1 (8%) | 3 (25%) | 0 (0%) | 0 (0%) | 4 (33%) |
| Any liver AEs | 2 (17%) | 4 (33%) | 2 (17%) | 1 (8%) | 4 (33%) |
|  |  |  |  |  |  |
| Non-haematological |  |  |  |  |  |
| Hypertension | 0 (0%) | 0 (0%) | 0 (0%) | 0 (0%) | 0 (0%) |
| Lethargy | 0 (0%) | 2 (17%) | 0 (0%) | 0 (0%) | 2 (17%) |
| Fatigue | 4 (33%) | 4 (33%) | 2 (17%) | 0 (0%) | 10 (83%) |
| Rash | 3 (25%) | 2 (17%) | 0 (0%) | 0 (0%) | 5 (42%) |
| Anorexia | 3 (25%) | 2 (17%) | 0 (0%) | 0 (0%) | 5 (42%) |
| Nausea | 4 (33%) | 2 (17%) | 0 (0%) | 0 (0%) | 6 (50%) |
| Vomiting | 0 (0%) | 1 (8%) | 0 (0%) | 0 (0%) | 1 (8%) |
| Constipation | 4 (33%) | 1 (8%) | 0 (0%) | 0 (0%) | 5 (42%) |
| Diarrhoea | 4 (33%) | 0 (0%) | 0 (0%) | 0 (0%) | 4 (33%) |
| Oedema | 4 (33%) | 3 (25%) | 0 (0%) | 0 (0%) | 7 (58%) |
| Allergy reaction | 1 (8%) | 0 (0%) | 0 (0%) | 0 (0%) | 1 (8%) |
| Tinnitus | 0 (0%) | 0 (0%) | 0 (0%) | 0 (0%) | 0 (0%) |
| Dyspnoea | 2 (17%) | 0 (0%) | 0 (0%) | 1 (8%) | 3 (25%) |
| Blurred vision | 3 (25%) | 0 (0%) | 0 (0%) | 0 (0%) | 3 (25%) |
| Other |  |  |  |  |  |
| Haemorrhage | 0 (0%) | 0 (0%) | 0 (0%) | 0 (0%) | 0 (0%) |
| Bacteraemia | 0 (0%) | 0 (0%) | 0 (0%) | 0 (0%) | 0 (0%) |
| Fever | 1 (8%) | 0 (0%) | 0 (0%) | 0 (0%) | 1 (8%) |
| Mucositis/oral thrush | 3 (25%) | 0 (0%) | 0 (0%) | 0 (0%) | 3 (25%) |
| Other mucositis | 0 (0%) | 0 (0%) | 0 (0%) | 0 (0%) | 0 (0%) |
| Paronychia | 0 (0%) | 0 (0%) | 1 (8%) | 0 (0%) | 1 (8%) |
| General infection | 0 (0%) | 0 (0%) | 3 (25%) | 0 (0%) | 3 (25%) |
| Biliary sepsis | 0 (0%) | 0 (0%) | 0 (0%) | 0 (0%) | 0 (0%) |
| Sensory neuropathy | 1 (8%) | 0 (0%) | 0 (0%) | 0 (0%) | 1 (8%) |
| Thromboembolic event | 0 (0%) | 0 (0%) | 0 (0%) | 0 (0%) | 0 (0%) |
| Chest pain - cardiac | 0 (0%) | 0 (0%) | 1 (8%)³ | 0 (0%) | 1 (8%) |
| Non-specific pain | 1 (8%) | 1 (8%) | 0 (0%) | 0 (0%) | 2 (17%) |
| Pancreatitis | 0 (0%) | 1 (8%) | 0 (0%) | 0 (0%) | 1 (8%) |
| Alopecia | 0 (0%) | 0 (0%) | 0 (0%) | 0 (0%) | 0 (0%) |
| Heart failure | 0 (0%) | 0 (0%) | 1 (8%) | 0 (0%) | 1 (8%) |
| Nasal discharge/congestion | 2 (17%) | 0 (0%) | 0 (0%) | 0 (0%) | 2 (17%) |
| Dyspepsia/Dysphagia | 0 (0%) | 1 (8%) | 0 (0%) | 0 (0%) | 1 (8%) |
| Retinal vascular disorder | 0 (0%) | 1 (8%) | 0 (0%) | 0 (0%) | 1 (8%) |
| Depressive symptoms | 0 (0%) | 0 (0%) | 0 (0%) | 0 (0%) | 0 (0%) |
| Palmar-plantar erythema | 0 (0%) | 0 (0%) | 0 (0%) | 0 (0%) | 0 (0%) |
| Other adverse events | 3 (25%) | 1 (8%) | 0 (0%) | 0 (0%) | 4 (33%) |
| Any non-haem AEs | 11 (92%) | 7 (58%) | 7 (58%) | 1 (8%) | 11 (92%) |
|  |  |  |  |  |  |
| ^1^ One patient can appear in more than one row | | | | | |
| ^2^ Percentages are based on a total of 12 patients. Patient 7 did not start treatment | | | | | |
| ^3^ This was considered a DLT (Patient 9) | | | | | |
| * Selumetinib related toxicity when classified as "Definitely", "Possibly" or "Probably" | | | | | |
