# Supplementary material for: A phase 1b study of Selumetinib in combination with Cisplatin and Gemcitabine in advanced or metastatic biliary tract cancer: the ABC-04 study
Source: BMC Cancer. 2016 Feb 24;16:153. doi: 10.1186/s12885-016-2174-8 (PMC4766710; doi:10.1186/s12885-016-2174-8)
Supplement: Additional file 3: Table S4. — Best objective response (RECIST v1.1). (DOCX 11 kb) [file 12885_2016_2174_MOESM3_ESM.docx]

| Table S4 -Best objective response (RECIST v1.1) | |
| --- | --- |
| Best Objective response (RECIST v1.1) | Selumetinib  75 mg bd (N%) |
|  |  |
| Best Objective response |  |
| Complete response | 1 |
| Partial response | 1 |
| Stable disease | 6 |
| Not evaluable* | 5 |
|  |  |
| Total | 13 |
|  |  |
