# Supplementary material for: A phase 1b study of Selumetinib in combination with Cisplatin and Gemcitabine in advanced or metastatic biliary tract cancer: the ABC-04 study
Source: BMC Cancer. 2016 Feb 24;16:153. doi: 10.1186/s12885-016-2174-8 (PMC4766710; doi:10.1186/s12885-016-2174-8)
Supplement: Additional file 4: Table S3. — Missing timepoints. (DOCX 10 kb) [file 12885_2016_2174_MOESM4_ESM.docx]

Table S3: missing timepoints

These are the timepoints with missing data. All the other timepoints are available.

patient 3 – sample 15 min and at 24h not done

patient 5 - missing time selumetinib taken

patient 6 - missing 24h sample

patient 8 - Missing time selumetinib taken

patient 9 - Patient took at home, time of selumetinib unreliable, missing times, this patient was removed from the analysis

patient 12 - sample at 24 hours missing
